# Host age alters amphibian susceptibility to *Batrachochytrium dendrobatidis*, an emerging infectious fungal pathogen

**DOI:** 10.1371/journal.pone.0222181

**Published:** 2019-09-06

**Authors:** Paul W. Bradley, Paul W. Snyder, Andrew R. Blaustein

**Affiliations:** 1 Environmental Sciences Graduate Program, Oregon State University, Corvallis, Oregon, United States of America; 2 Department of Integrative Biology, Oregon State University, Corvallis, Oregon, United States of America; University of South Dakota, UNITED STATES

## Abstract

Parasites and pathogens are often aggregated in a minority of susceptible hosts within a population, with a majority of individuals harboring low infection intensities. However, determining the relative importance of host traits to explain this heterogeneity is a challenge. One ecologically important pathogen is *Batrachochytrium dendrobatidis* (Bd), which causes the disease chytridiomycosis and has been associated with many amphibian population declines worldwide. For many hosts, post-metamorphic stages are generally more susceptible than the larval stage. Yet, examination of the effects of Bd infection at different ages within a life stage, has received little attention. This study investigated the hypothesis that recently-post-metamorphic frogs were more sensitive to chytridiomycosis than older frogs, and that sensitivity to Bd infection decreased as frogs aged. We examined this relationship with Pacific treefrogs (*Pseudacris regilla*) and red legged frogs (*Rana aurora*). Age had a strong effect on susceptibility to infection, infection intensity, and survival–but not in the directions we had predicted. In both host species, an increase in age was associated with frogs becoming more susceptible to Bd infection, harboring larger infection intensities, and greater risk of mortality. This suggests that the timing of Bd exposure may influence amphibian population dynamics.

## Introduction

Heterogeneity in host infection status and infection intensity is well documented in numerous disease systems [1–4]. Parasites and pathogens are generally aggregated in a minority of susceptible hosts within a population, with a majority of individuals harboring low infection intensities [5]. This distribution can result from differences in host risk of exposure, or susceptibility to infection after exposure [6]. Risk of exposure can be a function of environmental variation across the landscape and/or can occur via variation in host traits such as intrinsic susceptibility, vigor, sex, behavior, or age. However, determining the relative strength of host traits to determine the outcome of infection can be challenging. This is particularly true of host age. Age-related changes in infection prevalence can have several different causes [4, 7]. Infection prevalence can increase as a host ages, due to a longer period of exposure to the pathogen or due to increased numbers of host-host contacts over time [8]. Infection prevalence can decrease as a host ages due to differential survival between infected and uninfected individuals in the population, resulting in an elevated proportion of uninfected survivors [9]. Infection prevalence can also decrease due to acquired immune responses or age-related changes in host behavior that decrease risk of exposure or infection [10]. Assessing how infection dynamics differ across host ages is important to accurately predict infection patterns that may help mitigate population declines or extirpations associated with wildlife diseases.

One disease that has been associated with numerous population declines worldwide is chytridiomycosis. This disease is caused by a fungus, *Batrachochytrium dendrobatidis* (Bd), which infects numerous amphibian species [11, 12]. However, susceptibility to Bd varies with species [13–17], population [18, 19], life stage [20, 21], Bd strain [22, 23], and survival after exposure to Bd is often context dependent varying with a variety of co-factors [24–28]. Many species of frogs appear to exhibit an elevated susceptibility to chytridiomycosis immediately following metamorphosis [20, 29–31], suggesting that metamorphs may be more at risk for Bd infection than other age groups [19, 32–34], however there is a paucity of experimental research addressing the effect of post-metamorphic age on the susceptibility to infection with Bd [30, 35–37]. In this study, we investigated how susceptibility to Bd infection, and the disease chytridiomycosis, varied across the first nine months post-metamorphosis, specifically testing the hypothesis that frogs are most sensitive to Bd immediately after metamorphosis, and that older post-metamorphic frogs are less susceptible.

Over the first year of growth following metamorphosis, one would expect age to be correlated with body size [38]. As such, if host susceptibility to Bd infection were dose dependent, then an inoculum made up of a fixed dose of Bd zoospores regardless of frog age would decrease in relative strength over the experiment as the individual frogs increased in size/age [39]. Thus an additional Bd exposure treatment would be necessary–a mass-specific dose calculated to be a constant inoculation relative to body size as the frogs grew.

## Materials and methods

### Study species and sites

To test our hypothesis experimentally, we used two species of anuran amphibians: the Pacific treefrog (*Pseudacris regilla*) and the red legged frog (*Rana aurora*). These species were selected because both have been observed in the field with Bd infections [16, 40, 41], and both species are susceptible to chytridiomycosis with death as a potential outcome after infection [14, 42]. Furthermore, it is common for these two species to undergo metamorphosis at approximately the same time in the Oregon Willamette Valley, where we collected eggs of this species, allowing both species to be investigated simultaneously in a comparative experimental regime. The study was performed using individuals collected as eggs, reared under uniform conditions through metamorphosis and post-metamorphic ages.

In year one, to ensure Bd-naïve animals, all individuals utilized in the experiment were collected as eggs, which cannot be infected by Bd [43]. Pacific treefrog eggs were collected from >20 clutches found in a temporary pond near Corvallis, OR (Benton County, elevation 93 m; latitude/longitude: 44.572/-123.301) and red legged frog eggs were collected from >10 clutches located in a permanent pond located near Florence, OR (Lincoln County, elevation 12 m; latitude/longitude: 44.088/-124.123). For both species, eggs were collected the same day (11 February 2012) and consisted of early-stage embryos [44]. Immediately after collection, eggs were transported to Oregon State University where they were placed in 40-L aquaria filled with dechlorinated water. Upon hatching larvae were kept at a density of approximately 200 individuals per aquarium and fed a mixture (1:3 ratio by volume) of Tetramin fish food and ground alfalfa pellets *ad libitum*. Aquaria were kept at 14° C, under a natural photoperiod, and water was changed every seven days.

Animals used in this experiment were collected under a permit issued by the Oregon Department of Fish and Wildlife and animals were cared for in accordance with guidelines provided in the Guide for the Care and Use of Laboratory Animals [45] and all efforts were made to minimize suffering. The research was reviewed and approved by Oregon State University IACUC animal care and use permit 4340.

### Mesocosm experimental setup

On 27 May 2012, at Gosner [44] stage 25, 250 larval tadpoles of each species were transported to outdoor mesocosms at the Lewis Brown Horticulture Farm near Corvallis, (Benton County, Oregon; elevation 71 m). Mesocosms were 0.9 m in diameter and filled to a depth of 0.4 m with approximately 322 L of well water. To establish a natural microbial aquatic community, mesocosms were inoculated with 1 L of pond water collected from nearby wetlands, 100 oak (*Quercus* spp.) leaves, 50 g of alfalfa pellets, and allowed to sit for 30 days before the addition of larvae. Individuals of each species were equally divided among five mesocosms with a density of 50 individuals per mesocosm. Larvae were checked weekly until they reached Gosner [44] stage 41 (at least one forelimb visible), and then were checked daily. Individuals completed metamorphosis between 9 July to 15 August 2012. Upon metamorphosis frogs were moved from aquatic mesocosms to terrestrial mesocosms (0.9 m wide x 0.7 m across x 0.3 m deep). Terrestrial mesocosms were located adjacent to the aquatic mesocosms, lined with wet sphagnum moss. Newly metamorphosed frogs were fed pinhead crickets *ad libitum*. A mesh screen covered both the aquatic and terrestrial mesocosms to keep predators out and to keep frogs from escaping. After completing metamorphosis, frogs were transported back to Oregon State University and kept in 40-L glass terraria. All individuals were kept at a constant 14° C and 12:12d (light:dark) photoperiod. To limit the role that decreasing animal density might potentially play over the length of the nine-month study, frog density was limited to approximately 30 individuals per holding terrarium at all times. Individuals in the first year were allowed to acclimate to the laboratory environment for 7-days before the start of the first trial.

### Bd culture preparation

Ten days prior to the start of each trial, 1% tryptone agar Petri dishes for use in the upcoming trial were inoculated with liquid Bd culture (Bd strain JEL 274) and incubated at 22° C. This inoculation occurred concurrently with the passage of the culture into a new beaker of 1% tryptone broth. Thus, for each trial, the culture used to inoculate the Petri dishes had been re-passaged approximately 60 days prior. To harvest the zoospores at the start of each trial, Petri dishes were flushed with 15 mL of 22° C dechlorinated water and remained undisturbed for 5 minutes. The dishes were scraped with a rubber spatula to release the zoospores and sporangia adhering to the agar. The inoculum from each dish was then pooled in a beaker and the number of moving zoospores was determined using a hemocytometer. After quantifying the zoospore concentration, the inoculum was diluted to either 10,000 zoospores/mL for the fixed dose or to the calculated mass-specific dose (S1 and S2 Tables).

### Bd DNA extraction and qPCR

Following animal death and preservation, we used quantitative polymerase chain reaction (qPCR) to quantify Bd-infection intensity of all individuals in the Bd-exposure treatments. Additionally, we investigated Bd-infection status in two unexposed individuals per species per trial as well as every unexposed individual that died during the trials. To sample the individuals for Bd, we used a sterile, fine-tipped, dry swab (Medical Wire and Equipment, Corsham, Wiltshire, England) and swabbed the right ventral surface of individual frogs 10 times including the feet, legs, and drink patch. Individual swabs were placed into sterile screw-capped vials. Bd-DNA was extracted by adding 60 μL of Prepman Ultra (Applied Biosystems, Carlsbad, California), heating the vial for 10 min at 100° C, cooling the vials for 2 min, obtaining the supernatant, then diluting it to a 10% solution. We conducted qPCR using an ABI PRISM 7500 sequencer (Applied Biosystems) according to the methods of Boyle et al. [46]. All samples were run in triplicate and averaged. If a sample tested positive for Bd-DNA in only one replicate we reanalyzed the sample. If a second analysis was required, we re-swabbed the individual on their left side and analyzed the sample from the second swabbing.

### Coarse-scale differences in age investigating older post-metamorphic frogs

The first year of the experiment consisted of five age trials run consecutively. Each trial occurred 60-days after the previous trial and each trial with Bd-naïve frogs 60 ± 7.5 days older than in the previous trial (1-, 3-, 5-, 7-, 9-months post-metamorphosis). The 60-day difference in age between trials was selected in response to the number of individual frogs available to us at the start of the study and the availability of the necessary laboratory equipment and space.

At the start of each trial, all available Bd-naïve individuals were placed individually in beakers that had been randomly numbered. Then 36 of these individuals of each species were randomly selected from the pool. Frog mass was collected from each individual. For each species, the average mass was then calculated, and each frog was placed into an individual 600-mL (12.5 cm x 9 cm round) glass beaker, where it was housed for the duration of the trial. Each beaker had a mesh screen secured to the top to provide air circulation into the beaker but prohibit the escape of frogs. The 36 individuals in each trial were randomly assigned to and equally divided among, one of three exposure treatments: (a) a fixed dose of Bd zoospores (n = 12), (b) a mass-specific dose of Bd zoospores (n = 12), or (c) an unexposed control treatment (n = 12). The fixed dose of Bd zoospores consisted of 10 mL of inoculum containing 10,000 zoospores/mL for a total of 100,000 zoospores. The mass-specific dose consisted of 10 mL of inoculum containing a concentration of zoospores calculated at 5,000 zoospores multiplied by the average mass (in grams) of all 36 individuals in that trial (S1 and S2 Tables).

Individuals in the Bd-exposed treatments were exposed to 10 mL of inoculum poured directly on their dorsal surface while they were housed in beakers. This volume of inoculum was sufficient to cover the bottom of the beaker with a thin film. Control individuals were exposed to 10 mL of sham inoculum lacking the Bd culture, (made from 1% tryptone sterile agar plates following the same methods) poured onto their dorsal surface. The first trial began on 11 August 2012 with the initiation of subsequent trials every 60 days.

Over each 15-day trial, survival, frog-position (bottom versus side of beaker) and water level within the beaker were monitored daily. To maintain a thin film of water covering the entire bottom of a beaker, 5 mL of 14° C dechlorinated water was added daily to each beaker if necessary; no beaker was allowed to dry out before adding more water. Individuals were fed crickets (*Acheta domestica*) on the fifth, ninth, and 14th day and the water was changed in the beaker on the ninth day. The frogs utilized in the first year were fed 6 crickets; individuals in the one- and three-month post-metamorphosis trials were fed one-week old crickets, and individuals in the five- seven- and nine-month post-metamorphosis trials were fed two-week old crickets. Animals that died during the trial were preserved in 95% ethanol. Individuals that survived until the end of the trial (i.e. day 14) were euthanized in a 2% solution of MS-222, and then preserved in 95% ethanol.

### Fine-scale differences in age investigating younger post-metamorphic frogs

As we became aware our observations in the first year (ages 1-, 3-, 5-, 7-, and 9-months post-metamorphosis) were counter to our prediction we decided to run another set of trials using even younger frogs. Thus, while originally planned for only one year, the experiment was conducted for two years. The second year of the experiment consisted of three age trials run concurrently. Each trial comprised of Bd-naïve frogs that differed in post-metamorphic age by approximately one week (1-, 2-, 3-weeks post-metamorphosis). This difference in age was selected to capture potential age-related differences within individuals more recently experiencing metamorphosis than the youngest individuals utilized in the previous year.

Methods were the same as in Year 1 except for the following. Eggs were collected on 15 February 2013 (red legged frog) and 8 March 2013 (Pacific treefrog) and tadpoles were transported to the outdoor mesocosms on 20 May 2013. Metamorphic climax occurred between 3 and 18 July 2013. After moving the post-metamorphic frogs from the mesocosms to the laboratory, trials began after individuals acclimated for two days. Due to the unpredictable timing of metamorphosis, sample sizes differed in the first trial investigating Pacific treefrogs nearest to metamorphosis (S1 Table). All three age trials in the second year were run concurrently with all individuals among all age classes undergoing the same exposure procedure on the same day (20 July 2013). Thus, controlling for a change in density within holding terraria between the ages was not required. As in the previous year, the frogs utilized in the second year were fed on the fifth, ninth, and 14th days of the trials, and due to the relatively small body size of recently-post metamorphic frogs they were fed four pinhead crickets.

### Statistical analyses

We performed statistical analysis using TIBCO Spotfire S+ version 8.1 for Windows. We used logistic regression to test the prediction that infection prevalence in Bd-exposed individuals was negatively associated with age. The most parsimonious model explaining the odds of an individual becoming infected and maintaining an infection had the following explanatory variables: Bd exposure treatment (fixed vs. mass-specific), species, age, survival, and a Bd exposure treatment by species interaction.

Infection intensity values were log transformed (log-mean genome equivalents per individual + 1) prior to analysis to meet the normality assumptions required for statistical analysis. To test the prediction that infection intensity of infected individuals was negatively associated with age, we used a generalized linear model. The model was selected after performing a backwards stepwise comparison process starting with the following explanatory variables: species, Bd-exposure treatment, survival, mass, age, post-exposure day of death, and proportion of observations with individual on the side of the beaker. After investigating the variance inflation factor and performing a simple linear regression, frog mass was determined to be highly correlated with age and was removed from the model. The most parsimonious model had the following explanatory variables: species, Bd-exposure treatment, survival, and age.

We used a Cox proportional hazards model to investigate survival differences. The final model was selected via a backwards stepwise process that included the following explanatory variables in the full model: a binary indicator for exposure to Bd, specific exposure treatment, species, age, and mass in the model. The most parsimonious model lacked mass as an explanatory variable and thus we compared survival between the three exposure treatments (sham control vs. fixed vs. mass-specific) and two host species, as well as to test our prediction that risk and rates of mortality after Bd exposure would decrease as hosts aged. We performed Kaplan-Meier analyses to generate survival curves allowing us to visualize the differences in survival.

## Results

Infection after Bd exposure was not consistent across host ages (Table 1). Infection prevalence increased with age (χ^2^ = 4.89, *df* = 1, p = 0.027) and an individual was 5.8% more likely to become infected with each 60-day increase in age. However at any given age, the likelihood of obtaining an infection after exposure did not differ between species (χ^2^ = 0.212, *df* = 1, p = 0.6) or between the fixed dose and mass-specific dose Bd-exposure treatments (χ^2^ = 0.246, *df* = 1, p = 0.6). None of the individuals in the control treatment were infected. However all individuals in the Bd-exposed treatments that died were infected and mortality before the end of a trial was associated with an increase in the likelihood of becoming infected (χ^2^ = 13.3, *df* = 1, p < 0.001) when compared to survivors of the same age.

**Table 1:** Proportion infected individuals in each of the Bd-exposure treatments.

	Pacific treefrog (*Pseudacris regilla*)			Red legged frog (*Rana aurora*)		
Post-metamorphic Age	Proportion Infected After Fixed Dose Treatment	Proportion Infected After Mass-Specific Dose Treatment	Difference between the Mass-Specific and Fixed Dose Exposure Concentrations (zoospores/mL)	Proportion Infected After Fixed Dose Treatment	Proportion Infected After Mass-Specific Dose Treatment	Difference between the Mass-Specific and Fixed Dose Exposure Concentrations (zoospores/mL)
1-week	0.9091	0.8182	-7,652	0.6667	0.9167	-5,740
2-weeks	1	1	-7,422	1	0.6667	-5,160
3-weeks	0.9167	1	-7,359	0.9167	0.75	-4,760
1-month	0.6667	0.75	-6,658	0.9167	0.8333	-5,420
3-months	0.8333	0.8333	-5,041	1	1	-2,990
5-months	0.8333	0.9167	-2,864	1	1	1,092
7-months	0.9167	1	-2,637	0.8333	0.75	1,180
9-months	1	1	-2,867	1	0.9167	1,219

The proportion infected of Pacific treefrogs (*Pseudacris regilla*) and red legged frogs (*Rana aurora*) after exposure to the fixed dose treatment (10,000 zoospores per mL) or the mass-specific dose treatment, and the difference in concentration of Bd zoospores per mL between the two Bd exposure treatments for each trial. Trials one through three were performed on frogs that differed in post-metamorphic age by approximately one week (1-, 2-, 3-weeks post-metamorphosis). Trials four through eight were performed on frogs that differed in post-metamorphic age by approximately 60-days (1-, 3-, 5-, 7-, 9-months post-metamorphosis).

**Fig 1:**
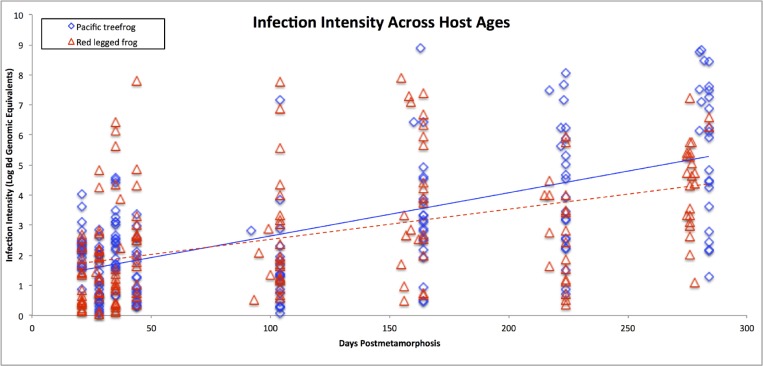
*Batrachochytrium dendrobatidis* (Bd) infection intensity measured at death over eight trials. Bd exposure trials were performed on frogs that differed in age by approximately one week (1-, 2-, 3-weeks post-metamorphosis) through the first month post-metamorphosis, then on frogs that differed in age by approximately 60-days (1-, 3-, 5-, 7-, 9-months post-metamorphosis).

Host age was positively associated with infection intensity (*t*_336_ = 6.23, p < 0.001) when controlling for host species, Bd-exposure treatment, and survival outcome (Fig 1). Each additional 60-day increase in age was associated with an increase in median infection intensity by 36.6% (95% CI of 24%-48%). Host species was a significant predictor of infection intensity (*t*_336_ = 4.89, p < 0.001). Red legged frogs displayed 44.9% lower median intensities (95% CI of 12%-77%) than Pacific treefrogs when holding the other predictors constant. However, the particular Bd-exposure treatment (fixed dose or mass-specific dose) was not associated with infection intensity (*t*_336_ = 1.92, p = 0.056). Survival outcome strongly predicted infection intensity at death (*t*_336_ = 12.1, p < 0.001). Individuals that survived the 15-day trial had lower median infection intensities by a factor of 19.2 (95% CI of 18.7–19.6).

**Fig 2:**
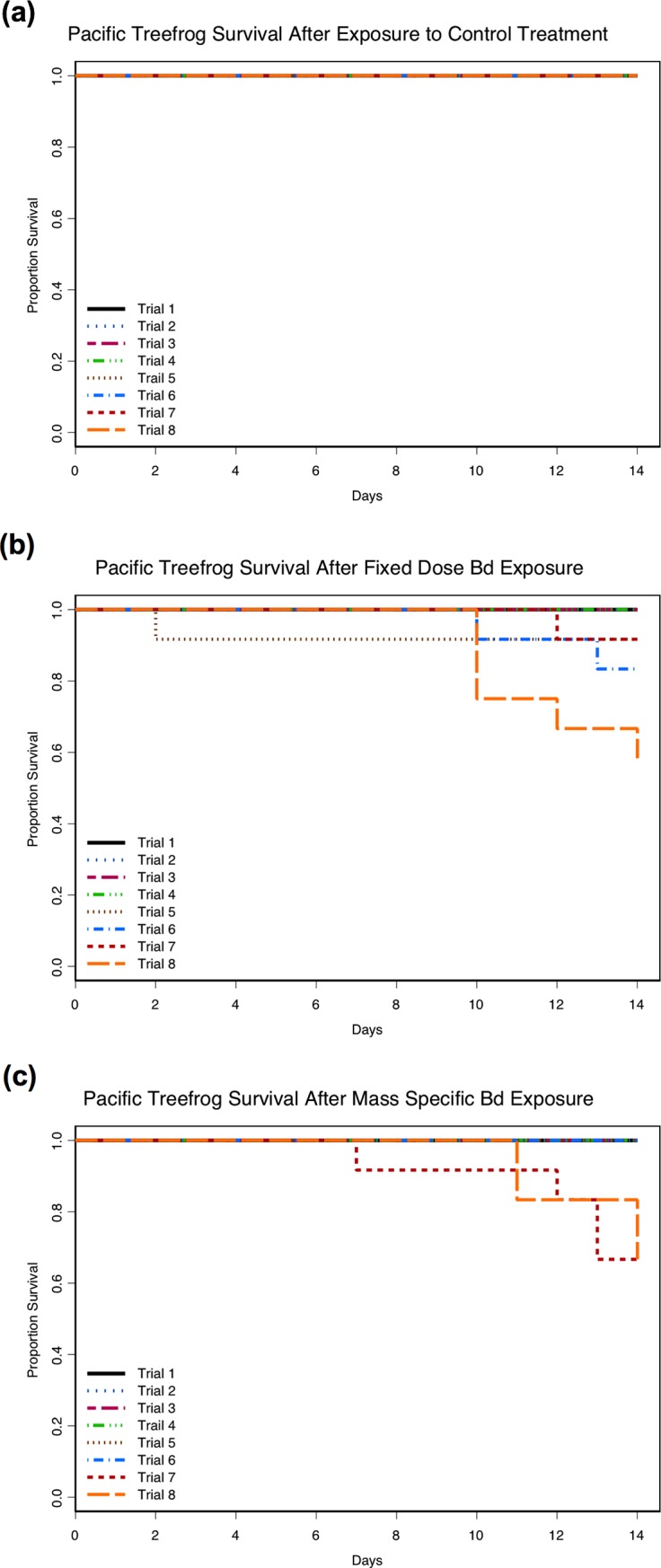
Survival among the eight age trials for Pacific treefrogs (*Pseudacris regilla*). Portion survival in the unexposed control treatment (a), after exposure to *Batrachochytrium dendrobatidis* (Bd) in the fixed dose exposure treatment (b), and after exposure to Bd in the mass-specific exposure treatment (c). Trials one through three were performed on frogs that differed in post-metamorphic age by approximately one week (1-, 2-, 3-weeks post-metamorphosis respectively). Trials four through eight were performed on frogs differed in post-metamorphic age by approximately 60-days (1-, 3-, 5-, 7-, 9-months post-metamorphosis respectively).

**Fig 3:**
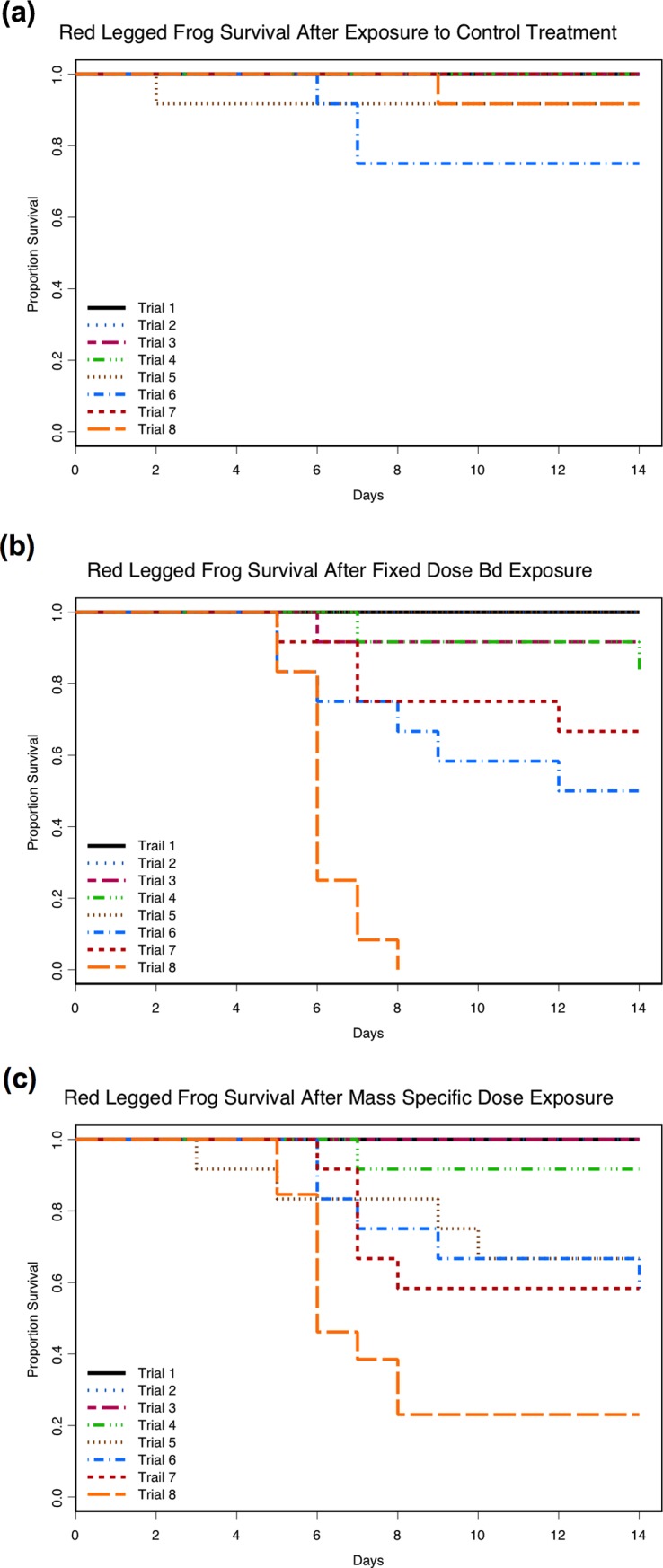
Survival among the eight age trials for red legged frogs (*Rana aurora*). Proportion survival in the unexposed control treatment (a), after exposure to *Batrachochytrium dendrobatidis* (Bd) in the fixed dose exposure treatment (b), and after exposure to Bd in the mass-specific exposure treatment (c). Trials one through three were performed on frogs that differed in post-metamorphic age by approximately one week (1-, 2-, 3-weeks post-metamorphosis respectively). Trials four through eight were performed on frogs differed in post-metamorphic age by approximately 60-days (1-, 3-, 5-, 7-, 9-months post-metamorphosis respectively).

More mortality occurred in the Bd-exposure treatments (Figs 2 and 3) with death occurring in 67/382 (17.5%) individuals exposed to Bd, compared with 5/190 (2.6%) unexposed control individuals (S3 and S4 Tables). The negative effect on survival after exposure to the pathogen was not consistent across ages or between host species. The risk of mortality after exposure to Bd differed between species (LR = 181, *df* = 4, p < 0.001) with 50/67 (74.6%) of the observed Bd-related mortality occurring in red legged frogs resulting in an increase in risk of mortality by a factor of 5.8 (95% CI of 3.3–10.1) compared to Pacific treefrogs (25.4% of observed Bd-related mortality) of the same age (Figs 2 and 3). The risk of mortality after Bd-exposure increased with age (LR = 181, *df* = 4, p < 0.001) for both amphibian species (Figs 2 and 3). Mortality in either of the Bd-exposed treatments was not observed until the third trial (3-weeks post-metamorphosis) for red legged frogs, or until the fifth trial (3-months post-metamorphosis) for Pacific treefrogs. For either species and for any given age, the risk of mortality after exposure to Bd did not differ between the two Bd exposure treatments (LR = 181, *df* = 4, p = 0.57).

## Discussion

Host age can play an important role in determining both the risk and outcome of pathogen exposure in many disease systems [47–49]. Infected hosts are often not uniformly distributed across ages of wild populations [50–52] as even small differences in host competence can result in a skewed distribution of parasites or pathogens [6]. Investigating the relationship between host age and disease in natural populations can be challenging. It can be difficult to tease apart the relative effect of aging alone, against the background of other host traits that change over time, such as the host mass, immune status, or age-associated changes in host behavior that may alter the risk of pathogen exposure.

In this laboratory study under constant and controlled environmental conditions, frog age had a strong effect on susceptibility to Bd infection, Bd infection intensity, and survival after exposure to the pathogen, but not in the directions we had predicted–in both amphibian species, an increase in age was associated with frogs becoming more susceptible to infection, maintaining elevated infection intensities, and greater proportions of mortality after exposure to the pathogen. This relationship between frog age and Bd infection prevalence did not differ between the two host species, nor did it differ between the two Bd-exposure treatments. Mortality during the 15-day trials was a strong predictor of positive infection status, and every individual that died during the trials tested positive for infection. Despite spending more time in the presence of the pathogen, survival until the end of the 15-day trial was, on average, associated with lower median infection intensity compared to individuals that died during the trial. Additionally, host species identity predicted infection intensities, with red legged frogs harboring lower intensities than Pacific treefrogs despite on average exhibiting a larger mass that we assumed would be associated with larger surface area for Bd to colonize.

As expected, exposure to the pathogen resulted in an increase in the risk of mortality. However, mortality was contingent upon age. As seen with infection susceptibility and infection prevalence, the specific Bd-exposure treatment did not result in differences in survival. The risk of mortality after exposure differed between the two host species; red legged frogs were more likely to die during the 15-day trial, despite harboring lower infection intensities. This suggests that differences in tolerance to Bd infection may exist between these two host species or alternatively that red legged frogs exhibit a greater ability to resist Bd infection than Pacific treefrogs but that this resistance is costly and might indirectly lead to frog mortality [53, 54].

For any given exposure dose, it would be reasonable to hypothesize that a larger surface area on older, and thus bigger, frogs might lead to a larger infection and be thus more likely to result in death [39]. And thus our observations of increased risk of mortality associated with age could be otherwise explained by the associated increase in body size. To address this potential outcome, we included a mass-specific Bd dose that remained constant across ages relative to the frog body size at any age. These two Bd exposure treatments differed in in Bd zoospore concentrations yet did not result in different infection intensities or risk of mortality. This held true despite the fixed dose treatment maintaining a total number of 100,000 zoospores regardless of frog mass, and the mass-specific dose treatment ranging between 23,000 to 71,000 zoospores for Pacific treefrogs and between 42,000 to 112,000 zoospores for red legged frogs (Table 1). These results imply that there is no dose effect within the range of Bd zoospore concentrations in this experiment for these two species of frogs. We did not observe differences in infection intensity or in mortality between the two Bd-exposure treatments, and thus interpret the observed differences in sensitivity to Bd to be due to an age-effect rather than a mass-effect. It is important to note however that the youngest frogs in our study were approximately 7-days post-metamorphosis and that it is possible that if we had explored Bd sensitivity during and immediately after metamorphic climax we might have observed the expected results.

The observed changes in sensitivity to Bd exposure as frogs aged could have been due to the post-metamorphic development of the frog immune system, which is not fully matured until well after metamorphosis [55, 56]. Recent studies show that both the adaptive branch of the frog immune system [57–60] and the innate immune system [61–66] may play a role in fighting Bd infection by resisting infection in a fast-responding and non-specific manner [67]. For example, host derived antimicrobial peptides (AMPs) secreted by the frog [63, 68, 69] and the natural microbiota of the frog skin [70–72] may prevent the colonization and infection of the skin by Bd. The production of a full suite of AMPs can take weeks after metamorphosis to develop [73].

It is also possible that the observed increases in susceptibility and sensitivity were due to post-metamorphic development outside of the immune system [74]. Mounting an immune response to pathogen exposure can be energetically costly [75] and any host must balance the energy demands of fighting an infection with the demands of other physiological and behavioral processes [76]. The relative cost of an immune response to infection is not constant and can change through development or as an environment changes. Therefore, both the relative cost of the immune response and the potential self-inflicted damage caused by an immune response could lead to greater host impairment [74, 77], but only after frogs have reached some host age or body size threshold necessary for a strong immune reaction to Bd infection [78].

Others have also observed low infection prevalence rates and the survival of infected recently-post-metamorphic Pacific treefrogs in both the laboratory and the field. In the laboratory Garcia et al. [79] showed that exposure to Bd did not increase mortality for recently-post-metamorphic Pacific treefrogs, whereas it did increase mortality in *Anaxyrus boreas* (western toads) and *Rana cascadae* (Cascades frog). Similarly, Searle et al. [21] showed that when larval Pacific treefrogs were exposed to Bd in outdoor mesocosms some individuals cleared the infection through metamorphosis. In field surveys, Piovia-Scott et al. [41] observed Bd infection prevalence to be lower in recently-post-metamorphic Pacific treefrogs than in adults and suggested that Bd growth rate might be limited in the Pacific treefrog at the younger ages. Our results are consistent with these studies and provide further evidence to suggest that the Pacific treefrog might be a tolerant carrier of Bd [16, 80]. However, the ability of the Pacific treefrog to tolerate high levels of infection may depend on frog age.

Investigators using both field and laboratory studies have observed heterogeneity in susceptibility to chytridiomycosis at different ages or stages. Mass mortality events of recently-post-metamorphic frogs have been reported while sympatric larvae of the same species survive nearby [29, 61, 81], implying heterogeneity in susceptibility across life-stages and elevated risk after metamorphosis. Similarly in the laboratory, Bd infected larvae often die soon after metamorphosis [20, 82, 83]. Likewise, field studies comparing frogs across post-metamorphic ages have also found elevated Bd infection prevalence and intensities in frogs of younger ages [31, 84, 85].

However, likely due to the time-consuming nature of such studies, fewer experiments have investigated the role of post-metamorphic age on the susceptibility to chytridiomycosis and the results have not been consistent. Lamirande and Nichols [30] observed survival in Bd-exposed sub-adult and adult Blue-and-yellow poison dart frogs, but observed 100% mortality within 32 days post exposure in recently-post-metamorphic frogs (7–14 days post metamorphosis at exposure). Ortiz-Santaliestra et al. [37] showed that American toads (*Anaxyrus americanus*) exposed to Bd 20 days after metamorphosis survived better than those exposed immediately after metamorphosis, however leopard frogs (*Lithobates pipiens*) showed no differences in survival between the two ages. And in a laboratory study investigating three age classes of green and golden bell frogs (*Litoria aurea*) Abu Bakar et al. [35] found a lower risk of morality with increasing age over the first 95 days after metamorphosis.

In conclusion, our results suggest that age can affect amphibian susceptibility to Bd infection. For both red legged frogs and Pacific treefrogs, the recently post-metamorphic age was the least susceptible to chytridiomycosis, with sensitivity to Bd increasing as frogs aged. This suggests that the timing of Bd exposure may influence amphibian population dynamics for susceptible species, and also suggests that individuals of a susceptible species can act as reservoirs for Bd as recently-post-metamorphic frogs, but may become susceptible hosts as they age. Thus, a deeper understanding the effect of post-metamorphic age on Bd infection is important for predicting the impacts of chytridiomycosis and managing imperiled amphibian populations.

## Supporting information

S1 Table Pathogen inoculation concentration in mass-specific dose treatment across all ages of Pacific treefrogs (*Pseudacris regilla*). The approximate post-metamorphic age of Pacific treefrogs (*Pseudacris regilla*) in each of the eight trials, the sample size for frogs in each exposure treatment and average mass for frogs, the average mass for frogs in each trial, and the concentration of *Batrachochytrium dendrobatidis* (Bd) zoospores in the mass-specific dose treatment for that trial.
(DOCX)

S2 Table Pathogen inoculation concentration in mass-specific dose treatment across all ages of red legged frogs (*Rana aurora*). The approximate post-metamorphic age of red legged frogs (Rana aurora) in each of the eight trials, the sample size for frogs in each exposure treatment and average mass for frogs, the average mass for frogs in each trial, and the concentration of Batrachochytrium dendrobatidis (Bd) zoospores in the mass-specific dose treatment for that trial.
(DOCX)

S3 Table Proximate cause of death in Pacific treefrogs that died during the 14-day trials (*Pseudacris regilla*). The approximate post-metamorphic age of Pacific treefrogs (*Pseudacris regilla*) in each of the eight trials, the sample size for frogs in each exposure treatment and the number of individuals that were euthanized during, that died (but not euthanized), during, and the number of individuals that survived until the end of, each trial.
(DOCX)

S4 Table Proximate cause of death in red legged frogs that died during the 14-day trials (*Rana aurora*). The approximate post-metamorphic age of red legged frogs (*Rana aurora*) in each of the eight trials, the sample size for frogs in each exposure treatment and the number of individuals that were euthanized during, that died (but not euthanized), during, and the number of individuals that survived until the end of, each trial.
(DOCX)

S1 Data Metadata and dataset collected in the study. (CSV)
