# Supplementary material for: Host age alters amphibian susceptibility to Batrachochytrium dendrobatidis, an emerging infectious fungal pathogen
Source: PLoS One. 2019 Sep 6;14(9):e0222181. doi: 10.1371/journal.pone.0222181 (PMC6730893; doi:10.1371/journal.pone.0222181)
Supplement: S1 Table — The approximate post-metamorphic age of Pacific treefrogs (Pseudacris regilla) in each of the eight trials, the sample size for frogs in each exposure treatment and average mass for frogs, the average mass for frogs in each trial, and the concentration of Batrachochytrium dendrobatidis (Bd) zoospores in the mass-specific dose treatment for that trial. (DOCX) [file pone.0222181.s001.docx]

S1 Table.
