# Supplementary material for: Host age alters amphibian susceptibility to Batrachochytrium dendrobatidis, an emerging infectious fungal pathogen
Source: PLoS One. 2019 Sep 6;14(9):e0222181. doi: 10.1371/journal.pone.0222181 (PMC6730893; doi:10.1371/journal.pone.0222181)
Supplement: S3 Table — The approximate post-metamorphic age of Pacific treefrogs (Pseudacris regilla) in each of the eight trials, the sample size for frogs in each exposure treatment and the number of individuals that were euthanized during, that died (but not euthanized), during, and the number of individuals that survived until the end of, each trial. (DOCX) [file pone.0222181.s003.docx]

S3 Table.
